# Critically appraised topic on adverse food reactions of companion animals (9): time to flare of cutaneous signs after a dietary challenge in dogs and cats with food allergies

**DOI:** 10.1186/s12917-020-02379-3

**Published:** 2020-05-24

**Authors:** Thierry Olivry, Ralf S. Mueller

**Affiliations:** 1 Department of Clinical Sciences, College of Veterinary Medicine, North Carolina State University, 1060 William Moore Drive, Raleigh, NC 27607 USA; 2 Medizinische Kleintierklinik, Centre for Clinical Veterinary Medicine, Ludwig Maximilian University, Veterinärstrasse 13, 80539 Munich, Germany

**Keywords:** Food allergy, Oral food challenge, Oral provocation, Relapse

## Abstract

**Background:**

At this time, elimination diets followed by oral food challenges (OFCs) represent the “gold standard” for diagnosing skin-manifesting food allergies (FA) in dogs and cats. Regrettably, there is no clear consensus on how long one should wait for clinical signs to flare after an OFC before diagnosing or ruling-out a FA in a dog or a cat.

**Results:**

We searched two databases on October 23, 2019 to look for specific information on the time for a flare of clinical signs to occur during OFCs after elimination diets in dogs and cats with skin-manifesting FAs. Altogether, we reviewed the study results of nine papers that included 234 dogs and four articles containing data from 83 cats. As multiple OFCs could be done in the same patient and not all animals included were subjected to an OFC, we were able to compile 315 and 72 times to flare (TTF) after an OFC in dogs and cats, respectively. When regrouping all cases together, about 9% of dogs and 27% of cats exhibited a flare of clinical signs in the first day after an OFC; 21% of dogs and 29% of cats had such relapse by the end of the second day. The time needed for 50 and 90% of dogs to exhibit a deterioration of clinical signs (TTF_50_ and TTF_90_) was 5 and 14, respectively; in cats, these times were 4 and 7 days, respectively. By 14 days after an OFC, nearly all food-allergic patients from both species had had a relapse of clinical signs. These results are limited by the likely under-reporting of flares that occur on the first day immediately following an OFC, the time in which IgE-mediated acute allergic reactions typically develop.

**Conclusion:**

Veterinary clinicians performing an OFC need to wait for 14 and 7 days for more than 90% of dogs and cats with a skin-manifesting FA to have a flare of clinical signs, respectively.

## Background

Cutaneous adverse food reactions (CAFRs), which are likely to represent food allergies (FAs) of immunologic origin, have a reported median prevalence of between 20 and 30% of pruritic, allergic or atopic dogs and half that in cats [1]. As, to date, we could not identify any published article reporting dogs and cats with non-immune CAFRs, we will use the term FA thereafter. In the canine and feline species, FAs exhibit a wide range of manifestations that are limited to the skin or the gut or that affect both organs (reviewed in [2, 3]. As FAs are not diagnosed reliably using laboratory procedures or in vivo tests [4], clinicians are left recommending the performance of lengthy elimination diets that involve the feeding of previously-uneaten ingredients or diets with sufficiently hydrolyzed protein sources [5]. By 8 weeks after starting such an elimination diet, the clinical signs of FA abate in over 90% of dogs and cats with FAs [5]. After documenting a marked improvement of clinical signs or a return to normalcy, ultimately confirming the diagnosis of FA will need the elicitation of a clinical flare after feeding the original diet, often followed by a subsequent abatement of signs with a further feeding of the elimination diet. A provocation test with single ingredients can be performed subsequently to identify those responsible for the allergic reaction. At this time, however, there is no clear consensus on how long veterinarians should wait before deciding that, because of a lack of recurrence of signs, a patient could be deemed non-allergic to the newly-fed item. Finding the shortest duration of time needed to rule-out an allergy to a (full) diet or its ingredients is of importance to help increase the owner’s compliance with this notoriously lengthy process, especially when performing sequential challenges to individual food components.

### Clinical scenario

A two-year-old male castrated Labrador retriever has a one-year history of chronic recurrent diarrhea and skin lesions consistent with atopic dermatitis. It is fed a salmon and potato-based commercial diet, and you thus suspect that it suffers from an allergy to this diet. You initiated an 8-week-long elimination diet with an extensive hydrolysate-containing diet, and cutaneous and digestive signs rapidly decreased in frequency and severity before eventually disappearing. The reintroduction of the salmon-and-potato diet resulted in a flare of signs within the first day. After signs abated again with the hydrolyzed diet, you confirmed a FA and you decided to identify which ingredient(s) caused this flare. You thus decided to first add salmon to the hydrolyzed diet. After 1 week, this dog was still is free of symptoms. You wonder if this short challenge duration was sufficient to rule-out a salmon allergy in this dog before proceeding with a second oral provocation with potatoes.

### Structured question

*In a dog or a cat with a skin-manifesting FA, what is the time needed for clinical signs to flare after an oral food challenge (OFC) with an ingredient to which it is allergic?*

### Search strategy

We searched the Web of Science Core Collection and CAB Abstract databases on October 23, 2019, with the following sensitive Boolean query string: (dog or dogs or canine or cat or cats or feline) and (food* or diet*) and (allerg* or hypersens*) not (human* or child*). There were no restrictions for publication dates or languages. We did not search conference abstracts or include review papers because of our need for original patient data. Finally, we scanned the bibliography of each selected article as well as those of previously-published critically-appraised topics on CAFRs in dogs and cats [1–7] for additional references.

### Identified evidence

Our query of the Web of Science and CAB Abstracts databases identified 489 and 877 articles, respectively*.* Among these, we searched for papers that reported specific information on the time for a flare of clinical signs to occur during the OFCs that followed elimination diets in dogs and cats with skin-manifesting FAs. For the purpose of this study, we considered OFCs made both with the original diet and its composing ingredients. Thus, we selected nine [8–16] and two [17, 18] papers relevant to dogs and cats with FAs, respectively. After scanning the bibliography of these articles and those of the previously published critically-appraised topics on CAFRs of companion animals [1–7], we identified three additional papers [19–21]. We subsequently eliminated one report [14] whose cases had been included in a second article published 2 years later [15]. Ultimately, the total number of articles selected was 13 of whom 11 were large case series or clinical trials [8–13, 15–18, 20] and the other two were case reports of one animal each [19, 21]. All but two studies involved dogs and cats with spontaneously-arising FAs occurring in a natural home environment, the last two were from dogs with spontaneous FAs who lived in a university-based laboratory animal facility [11, 13].

The patients included in the studies had a worldwide distribution, as five articles reported data from pets seen in the USA [9, 11, 13, 17, 19] two articles contained cases from the United Kingdom [10, 15] and France [8, 20] and there was one article from the Netherlands [12], Japan [21] and Australia [18]. The last paper included dogs from Switzerland and the USA [16]. The studies spanned nearly 30 years, as they were published between 1990 [8] and 2019 [16].

### Evaluation of evidence

Altogether, we reviewed the study results of nine papers that included 234 dogs and four articles containing data from 83 cats. As multiple OFCs could be done in the same patient or not all animals included were subjected to an OFC, we were able to compile 315 and 72 times to flare (TTF) after an OFC in dogs and cats, respectively. The full details are available in the Supplementary Tables 1 (dogs) and 2 (cats).

There was some variation in the reporting of TTF in the different studies. While the case reports [19, 21] and three of the large case series [10, 16, 20] specifically mentioned the TTF after OFCs in individual days, all other papers included TTF data in ranges two [8, 9, 11, 13, 18] to seven-day long [12, 17].

**Fig. 1:**
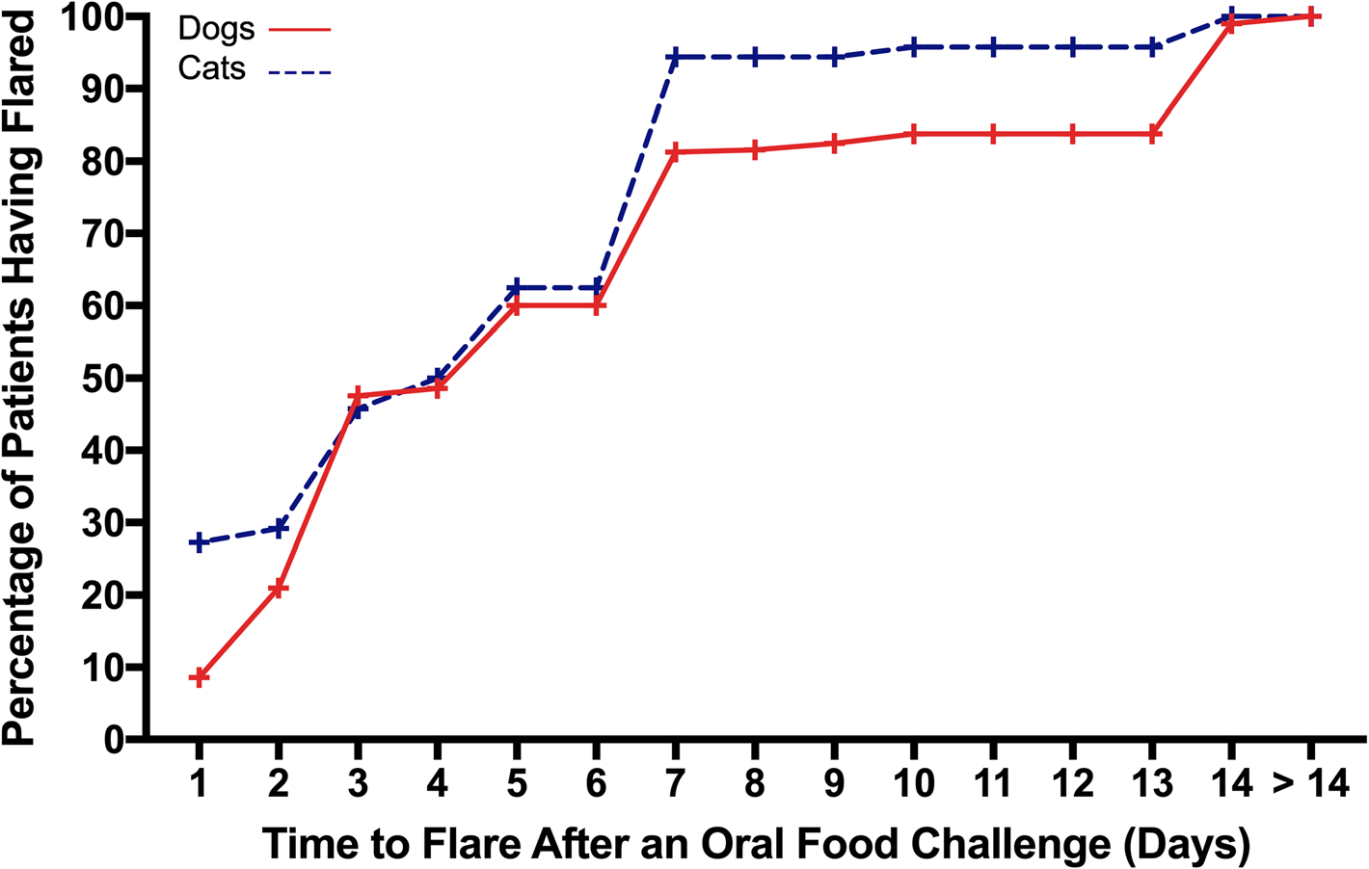
Cumulative daily probabilities of flares after an oral food challenge in dogs and cats with food allergies

When regrouping all cases together, the TTF after OFCs are depicted in Fig. 1. About 9% of dogs and 27% of cats exhibited a flare of clinical signs on the first day after an OFC; 21% of dogs and 29% of cats had such relapse by the end of the second day. The time needed for half of the dogs and cats to exhibit a deterioration of clinical signs (TTF_50_) was 5 and 4 days, respectively. Similarly, the TTF_80_ was 7 days for both species, and the TTF_90_ was 14 and 7 days for dogs and cats, respectively. By 14 days after an OFC, nearly all food-allergic patients from both species had had a relapse of clinical signs.

Two factors limit our interpretation of the data: 1) there was only a small number of animals (especially cats) with observations reported in the first day that follows an OFC, and, 2) several studies only included the TTF data in durations encompassing several days. Both factors likely led to an underreporting of TTF in the first hours or day after an OFC, the time when IgE-mediated acute allergic reactions typically are recognized.

### Conclusion and implication for practitioners

Veterinary clinicians performing an OFC need to wait for 14 and 7 days for more than 90% of dogs and cats with a skin-manifesting FA to have a flare of clinical signs (either skin lesions or pruritus), respectively. Despite the limitations highlighted above, the relatively low number of dogs and cats exhibiting a flare of cutaneous signs on the first day after an OFC suggest that, in these species, FAs might have more often a cell- rather than IgE-mediated pathogenesis [22]. This hypothesis could be one of the factors behind the low accuracy of food-specific IgE serological tests [4].

### Future research needs

Studies need to better document the flares that occur on the first day--especially in the first hours--after an OFC to establish the specific percentage of dogs and cats likely to have IgE-mediated FAs. Furthermore, as none of the papers reviewed herein provided sufficient details on the protocol of OFCs, there is a critical research need for the establishment of a standard OFC regimen with both commercial or homemade diets or single ingredients. In particular, the quantity of food provided, the type of diet (raw, cooked, a mix thereof) and the need, or lack thereof, for an escalating quantity and/or frequency of administration need to be investigated and harmonized.

## Supplementary information

**Additional file 1.** Supplementary Table 1 Data from studies reporting information from dogs with food allergies.

**Additional file 2.** Supplementary Table S2 Data from studies reporting information from cats with food allergies.

## Data Availability

All raw data included in this article are available in Supplementary Tables 1 and 2.

## Abbreviations

AD: Atopic dermatitis
CAFR: Cutaneous adverse food reaction
FA: Food allergy
OFC: Oral food challenge
TTF: Time to flare
